# Circadian metabolism in myeloid cells

**DOI:** 10.1093/lifemeta/loaf018

**Published:** 2025-05-29

**Authors:** Yingxi Xu, Chen Wang, Ping-Chih Ho

**Affiliations:** State Key Laboratory of Experimental Hematology, National Clinical Research Center for Blood Diseases, Haihe Laboratory of Cell Ecosystem, Institute of Hematology & Blood Diseases Hospital, Chinese Academy of Medical Sciences & Peking Union Medical College, Tianjin 300020, China; Tianjin Institutes of Health Science, Tianjin 301617, China; National Key Laboratory of Immunity and Inflammation, Suzhou Institute of Systems Medicine, Chinese Academy of Medical Sciences & Peking Union Medical College, Suzhou, Jiangsu 215123, China; Department of Oncology, University of Lausanne, 1066 Lausanne, Switzerland; Ludwig Institute for Cancer Research, University of Lausanne, 1066 Epalinges, Switzerland

**Keywords:** circadian rhythms, myeloid cell, metabolism

## Abstract

Circadian rhythms are fundamental regulators of physiological processes, including immune function. Recent insights uncover that not only lymphocytes but also myeloid cells possess intrinsic circadian clocks that govern their behavior and function. Emerging evidence highlights how circadian regulation of metabolism critically shapes the inflammatory and tissue-repair functions of myeloid subsets. Furthermore, mitochondrial dynamics, a key metabolic feature, are under circadian control and influence antigen presentation and effector functions. Here, we review the interplay between circadian clocks, metabolism, and myeloid immunity, discussing their therapeutic opportunities for optimizing vaccination, infection management, and immunotherapy.

## Introduction

The term “circadian” originates from the Latin *circa diem*, meaning “about a day” [1]. Mammalian circadian clocks are evolutionarily conserved systems that are entrained by rhythmic external cues, such as light exposure and food intake. This 24-h rhythm regulates a series of physiological processes, including cell division, metabolism, and immune surveillance, as well as pathological processes such as autoimmune disease, cardiovascular disease, and cancer.

Emerging studies underscore the clinical implications of circadian timing in therapeutic interventions. For instance, in allogeneic hematopoietic stem cell transplantation (allo-HSCT), the timing of stem cell infusion critically influences patient outcomes. Early-day infusions are associated with a reduced incidence and severity of acute graft-versus-host disease (aGVHD) and improved survival [2]. Similarly, circadian rhythms also shape antitumor immunity, with CD8⁺ T cell infiltration fluctuating according to the time of day, influenced by both immune cell and endothelial clocks. These rhythms affect the efficacy of therapies like the chimeric antigen receptor (CAR) T cell therapy and immune checkpoint blockade. Time-of-day-dependent T-cell signatures correlate with treatment response and survival, especially in melanoma, highlighting the potential of timing-based optimization in cancer immunotherapy [3].

Recent advances in circadian immunology have revealed intricate and bidirectional interactions between the immune system and the circadian clock, emphasizing that timing plays a crucial role in immune surveillance and response. Although the circadian regulation of lymphocyte trafficking is well established, its influence on myeloid cell function—particularly metabolic dynamics—remains less explored. This represents a critical gap, given the central role of myeloid cells in orchestrating both innate and adaptive immunity.

This review aims to highlight the significance of circadian rhythms in myeloid cell research and discuss future directions for integrating chrono-immunology into healthcare.

## Chronobiology

### Central clock and peripheral clock

In mammals, circadian rhythms are regulated by a central clock and peripheral clocks. The central clock, located in the suprachiasmatic nuclei (SCN) of the hypothalamus, receives light stimuli via the retina and the retinohypothalamic tract. Light stimulation synchronizes rhythmic activity within the SCN, the master clock. The master clock then coordinates the humoral and neural outputs to synchronize peripheral clocks in cells that do not receive direct light input.

The humoral output system operates through the hypothalamic-pituitary-adrenal (HPA) axis, in which the pituitary gland rhythmically secretes adrenocorticotropic hormone (ACTH), regulating the time-of-day-dependent release of steroid hormones and catecholamines from the adrenal gland. On the neuronal side, the sympathetic nervous system directly innervates peripheral organs, such as the bone marrow, spleen, and liver, contributing to the orchestration of peripheral rhythms. Beyond light, food intake can also reset circadian rhythms in specific tissues, such as the liver, which can become desynchronized from the master clock [4, 5]. The details of the central clock and peripheral clocks have been widely discussed in other reviews [1, 6].

Notably, many immune cells, such as macrophages and dendritic cells (DCs), possess functional, cell-autonomous molecular clocks [6]. While these internal oscillators are synchronized by systemic cues from the SCN, they are not entirely dependent on it [7, 8]. This integration enables immune cells to coordinate their local activities with broader systemic rhythms.

### Molecular clock

Circadian rhythms are a fundamental biological phenomenon observed across a wide range of species [9]. In mammals, these rhythms are regulated by a core molecular clock system built on a self-sustaining transcriptional–translational feedback loop. Key components include circadian locomotor output cycles kaput (*CLOCK*) and brain and muscle ARNT-like 1 (*BMAL1*, also known as *MOP3* and encoded by the *Arntl* gene). These transcription factors activate downstream genes such as period (*Per1/2*) and cryptochrome (*Cry1/2*), which in turn inhibit their own expression, forming a feedback mechanism that drives circadian oscillations [10].

**Figure 1:**
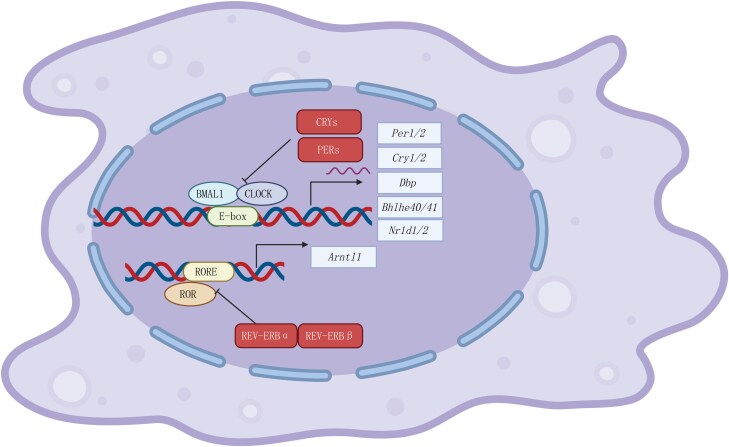
The molecular clock. The core clock molecules BMAL1 and CLOCK form a heterodimer that binds to E-box sequences in the promoter regions of clock-controlled genes (CCGs), driving their expression. This heterodimer also activates the transcription of negative regulators, including *Per* (period circadian protein), *Cry* (cryptochrome), and *Bhlhe40/41* (Dec1/2). The resulting PER–TRY complex subsequently inhibits the binding of the BMAL1–CLOCK heterodimer to E-boxes, creating a negative feedback loop. This repression continues until the levels of PER and CRY decline sufficiently to allow the cycle to restart. In addition to the PER–CRY feedback loop, a secondary regulatory cycle is maintained by REV-ERBα and REV-ERBβ (encoded by *Nr1d1* and *Nr1d2*, respectively). The REV-ERBα–REV-ERBβ complex competes with ROR for binding to ROR response elements (ROREs), thereby repressing the transcription of *Bmal1*. This intricate interplay of feedback loops ensures the precise regulation of circadian rhythms. Created with BioRender.com.

BMAL1 and CLOCK form a heterodimer that acts as a transcription factor. This complex binds to Enhancer (E)-box elements in the promoter regions of circadian genes, driving the expression of *Per1/2/3* and *Cry1/2*. The resulting PER and CRY proteins then assemble into a heterodimer that inhibits the BMAL1/CLOCK complex, thereby suppressing their own transcription. As PER/CRY levels decrease over time, BMAL1/CLOCK activity is restored, initiating a new circadian cycle. A secondary feedback loop, involving REV-ERB nuclear receptors-α/β (encoded by *Nr1d1/2*) and RAR-related orphan receptors (RORs), further stabilizes the molecular clock [6, 8] (Fig. 1).

### Zeitgeber time and circadian time

In chronobiology, Zeitgeber time (ZT) and circadian time (CT) are used to define experimental time references. A Zeitgeber (German for “time giver”) is an environmental cue that entrains circadian rhythms. ZT refers to the number of hours elapsed since the onset of the light phase. For example, in a standard 24-h light-dark (LD) cycle (12 h of light and 12 h of darkness), ZT is used as follows: ZT0/24 marks the onset of the light phase (“morning”), while ZT12 marks the beginning of the dark phase (“evening”). In contrast, under constant conditions such as continuous darkness (DD), CT is used, as there are no external entrainment cues. CT is a standardized 24-h time system based on the endogenous biological clock of the organism in the absence of external influences. CT0 represents the onset of the subjective day, corresponding to the time when lights should normally be turned on. Conversely, CT12 signifies the onset of the subjective night, when lights would normally be turned off.

## Circadian rhythms and myeloid cells

Circadian rhythms in the immune system were first reported in the innate immune system in 1960 [11]. Since then, studies have shown that leukocyte migration, cellularity, and function are regulated by circadian rhythms. Leukocyte levels in the bloodstream exhibit rhythmic fluctuations in both mice and humans, peaking during the rest phase—daytime for mice [12] and nighttime for humans [13].

Both systemic cues, such as glucocorticoids and sympathetic nervous activity, and intrinsic circadian machinery within leukocytes, particularly the core clock gene *Bmal1*, orchestrate immune rhythms [1]. Cell-specific knockout studies have demonstrated that deleting *Bmal1* disrupts the diurnal patterns of the respective immune cells, underscoring the presence of autonomous circadian regulation within these immune subsets [14].

Circadian rhythms in leukocytes are governed by both systemic signals, such as corticosteroids or sympathetic nervous system activity, and intrinsic molecular clock components like BMAL1. Tissue-specific knockout studies have substantiated the presence of autonomous circadian machinery within leukocytes, highlighting the intrinsic role of BMAL1 in regulating immune cell function. Studies have shown that circadian clocks regulate leukocyte migration and trafficking into tissues, ensuring dynamic control over their distribution at different times of the day [3, 12, 15–17]. In addition to migration, key leukocyte effector functions, including proliferation and cytokine production, also follow rhythmic patterns [18–21]. These immune system rhythms may have evolved to enhance defense, optimizing responses to potential pathogen encounters during periods of food intake and heightened behavioral activity in the active phase [22].

### Macrophages and monocytes

Among all immune cells, macrophages and monocytes are the most extensively studied cell types regarding circadian regulation. In addition to systemic influences, macrophages exhibit intrinsic circadian oscillations independent of SCN. Notably, *Bmal1* expression in peritoneal macrophages and bone marrow-derived macrophages (BMDMs) persists under constant darkness and in *ex vivo* conditions, confirming cell-autonomous rhythmicity [23]. The circadian genes such as *Bmal1* and *Per1/2* may control the development and function of macrophages. For example, deletion of *Per1* and *Per2* promotes a pro-inflammatory M1 macrophage phenotype in BMDMs [24], indicating that the circadian clock governs macrophage polarization between pro-inflammatory (M1) and anti-inflammatory (M2) states [24]. Moreover, the core clock gene *Bmal1* regulates the expression of toll-like receptor 4 (TLR4)-mediated inflammation in macrophages [25]. In a *Streptococcus pneumoniae* infection model, *Bmal1*-deficient macrophages exhibited enhanced bacterial clearance and reduced disease severity, further highlighting its role in phagocytosis [26]. Cytokine secretion, such as interleukin (IL)-1β, is also controlled by circadian rhythms. In macrophages, the CLOCK-BMAL1 heterodimer binds to the E-box elements of *NFE2L2* (NFE2-like BZIP transcription factor 2) promoter, a master antioxidant transcription factor, thereby suppressing IL-1β production [27]. Another clock gene, REV-ERBα (encoded by *Nr1d1*), regulates the diurnal expression of NLR family pyrin domain-containing protein 3 (NLRP3), a key component of the inflammasome pathway [28]. Mechanistically, REV-ERBα directly binds to the *Nlrp3* promoter, thereby suppressing IL-1β production [28]. Beyond IL-1β, REV-ERBα also controls the expression of IL-6 and C-C motif chemokine ligand 2 (CCL2) in macrophages [29, 30] and in microglia [31], highlighting its broader immune-modulatory roles.

Compared to macrophages, fewer studies have investigated the circadian regulation of monocytes. One major study found that Ly6C^high^ inflammatory monocyte recruitment to inflamed sites follows a time-of-day-dependent pattern [14]. Notably, *Listeria monocytogenes* infection at ZT8 (afternoon) resulted in reduced bacterial spread and stronger inflammatory responses than infections at ZT0 (morning), a difference associated with higher levels of IL-1β, tumor necrosis factor-alpha (TNF-α), interferon-gamma (IFN-γ), and CCL2 [14]. Chromatin immunoprecipitation (ChIP) analysis revealed direct BMAL1-CLOCK binding to the *Ccl2* promoter, confirming the transcriptional regulation of *Ccl2* by BMAL1 [14].

### Neutrophils

Circadian rhythms regulate neutrophil number and phenotype in a cell-autonomous manner [32]. The clock component BMAL1 controls the expression of C-X-C motif chemokine ligand 2 (*Cxcl2*), which interacts with C-X-C chemokine receptor type 2 (CXCR2) to drive neutrophil aging, i.e. a process by which neutrophils undergo functional and phenotypic changes over time after their release from the bone marrow into circulation [32]. NET (neutrophil extracellular trap) formation, which is crucial in neutrophil function, also appears to be regulated by circadian rhythms, as aged neutrophils release more NETs than younger ones [33]. A recent study also highlights the importance of neutrophils in forming the diurnal barrier of the skin [34].

Intriguingly, neutrophils have been implicated as potential “Zeitgebers” in different organs [15, 35]. Studies show that neutrophils gate the rhythmicity in the liver by infiltrating in a time-of-day-dependent manner [36]. During the light phase, neutrophils’ infiltration peaks, accompanied by increased neutrophil elastase (NE) release, which triggers *Bmal1* expression in hepatocytes via c-Jun NH2-terminal kinase (JNK) activation [36]. Similarly, in the intestines, neutrophil infiltration suppresses *IL-23* expression in resident macrophages, leading to reduced levels of granulocyte colony-stimulating factor (G-CSF) in circulation [37]. These findings underscore the circadian regulation of neutrophil behavior, which plays a more substantial role in organ function and immune homeostasis than previously recognized.

### DCs

DCs serve as antigen-presenting cells (APCs) that link the innate and adaptive immune systems. Recent studies underscore the pivotal role of circadian rhythms in modulating DC behavior and function. Following antigen uptake, DCs migrate to lymph nodes to present processed antigens to T cells. Recent studies reveal that DC entry into afferent lymphatic vessels occurs rhythmically, peaking at ZT7 (day) and reaching a nadir at ZT19 (night) [38]. During this process, chemokine CCL21 and adhesion molecules (lymphatic vessel endothelial hyaluronan receptor-1 (LYVE-1), CD99, and junctional adhesion molecule-A (JAM-A/F11R)) on lymphatic endothelial cells (LECs) act as key drivers of DC migration, with C-C motif chemokine receptor 7 (CCR7) on DCs further facilitating this process. *In vitro* synchronization of bone marrow-derived dendritic cells (BMDCs) confirmed that DC migration exhibits an intrinsic circadian rhythm [38].

A key study highlighted the intriguing diurnal function of DCs in a tumor model [39]. Researchers observed a higher number of DCs, including antigen-specific ones, in tumor-draining lymph nodes when tumors were inoculated during the day (ZT9). Further experiments demonstrated that DCs play a dominant role in the circadian regulation of anti-tumor immunity, influencing tumor growth control. This effect is dependent on the cell-intrinsic clock, as it is completely abolished in lineage-specific *Bmal1* knockout mice. Moreover, the costimulatory molecule CD80 on DCs exhibits circadian oscillation, directly regulated by the core clock molecule BMAL1, which binds to the promoter region of *Cd80*. Additionally, human monocyte-derived dendritic cells (Mo-DCs) display a similar rhythmic pattern of CD80 expression following synchronization, indicating a universal circadian-controlled pattern [39].

Circadian components REV-ERBα and REV-ERBβ also regulate DC maturation. *Ex vivo* studies demonstrated that loss of REV-ERBα or REV-ERBβ enhances DC activation, leading to increased CD86 and major histocompatibility complex class Ⅱ (MHCⅡ) expression and elevated *Il1b*, *Il6*, and *Il12b* levels [40]. Consistently, treatment with the REV-ERB agonist SR9009 suppressed DC maturation markers and inhibited pro-inflammatory cytokine production in BMDCs, indicating the anti-inflammatory effect of REV-ERBα, akin to its role in macrophages [27, 28].

The diurnal oscillation in DCs also contributes to the fate decision of lymphocytes. One study revealed that DC-induced expansion of antigen-specific CD8^+^ T cells follows a circadian oscillation, peaking around CT6 and dipping at CT18. This effect is partially attributed to intrinsic DC clocks, as *Bmal1* deletion in DCs impairs T cell migration to the spleen but does not completely abolish rhythmic expansion [41]. Moreover, circadian rhythms regulate the antigen processing of DCs, with enhanced T cell activation during the rest phase (ZT7) [42]. Mechanistically, *Bmal1* regulates mitochondrial fusion and function in a Ca^2+^-dependent manner, thereby controlling the antigen processing efficiency [42]. In murine tumor models, DCs exhibit circadian patterns that significantly influence anti-tumor immune responses. Specifically, tumor inoculation during the day (ZT9) results in a higher accumulation of DCs in the tumor-draining lymph nodes, leading to enhanced CD8^+^ T cell expansion [39]. Moreover, another study reported that the enhanced DC migration during the day elicits greater lymph node expansion, thus driving both T cell and B cell responses following vaccination [43].

## Circadian regulation of myeloid cell metabolism

The circadian clock not only governs immune cell activities like migration and cytokine secretion but also plays a critical role in regulating cellular metabolism. Emerging evidence indicates that metabolic pathways directly influence myeloid cell fate and function [44, 45]. Therefore, understanding how the circadian clock modulates metabolism offers important insights into its role in shaping myeloid cell behavior and immune responses.

### Macrophages: metabolic shifts between pro-inflammatory and anti-inflammatory phenotypes

Macrophage function and polarization are closely governed by their metabolic programming, particularly lipid, glucose, and amino acid metabolism. These pathways are pivotal in shaping either the pro-inflammatory M1 or anti-inflammatory M2 phenotype [45]. M1 macrophages depend on aerobic glycolysis, driven by hypoxia-inducible factor 1 subunit alpha (HIF1A) [46] and nuclear factor kappa B (NF-κB) [47], to rapidly generate adenosine triphosphate (ATP) and reactive oxygen species (ROS) for antimicrobial activity, especially in hypoxic environments [46]. Glucose uptake via glucose transporter-1 (GLUT1), also known as solute carrier family 2 member 1 (SLC2A1), enhances glycolysis and the pentose phosphate pathway, producing nicotinamide adenine dinucleotide phosphate (NADPH) for redox balance and ROS generation [48]. In contrast, M2 macrophages rely on fatty acid oxidation (FAO), fueled by lysosomal lipid breakdown, to support tissue repair and anti-inflammatory responses [49]. Amino acid metabolism also contributes significantly: arginine is converted by inducible nitric oxide synthase (iNOS) in M1 macrophages to nitric oxide for pathogen killing, while in M2 cells, arginase-1 processes arginine for tissue repair [50]. Glutamine and leucine further modulate polarization through energy supply and the mechanistic target of rapamycin (mTOR) activation [51, 52].

Circadian rhythms regulate the metabolic process of macrophages [53–55], which in turn regulates their fate decision and function. Several studies have demonstrated that circadian clock genes play a pivotal role in regulating macrophage lipid metabolism. For example, *Clock* mutant mice exhibit increased uptake of oxidized low-density lipoprotein (oxLDL), reduced cholesterol efflux in macrophages, and worsened atherosclerosis [56]. *Bmal1* and *Per1/2/3* knockout mice show enhanced uptake of oxLDL and cholesterol, as well as increased intracellular cholesterol ester accumulation in macrophages [57]. Circadian rhythms also influence macrophage glucose metabolism. Disruptions in circadian timing, such as *Bmal1* deficiency, lead to enhanced glucose uptake and glycolysis, resulting in increased lactate production, which is accompanied by reduced oxygen consumption and increased ROS production via succinate dehydrogenase in macrophages, thus promoting an M1-like inflammatory phenotype [58]. *Bmal1* also regulates the expression of glucose transporters and key glycolytic enzymes, including modulating both the abundance and subcellular localization of pyruvate kinase isoenzyme type M2 (PKM2). This modulation facilitates the activation of the signal transducer and activator of transcription 3 (STAT3), thereby enhancing *Il-1β* expression [59]. These metabolic shifts in macrophage activity elicited by disruptions in circadian timing contribute to chronic inflammation and metabolic disease. Moreover, it is well known that the circadian clock regulates amino acid metabolism, including the uptake and utilization of arginine, glutamine, and methionine [60]. Myeloid *Bmal1*-deficient macrophages exhibit enhanced amino acid metabolism due to upregulated amino acid transporters, leading to increased oxidative stress and activation of HIF1A. This, in turn, promotes an M2-like, immunosuppressive tumor-associated macrophage phenotype that supports tumor progression [58]. Circadian rhythms in circulating amino acid levels have been observed in both mice and humans, suggesting a broader regulatory mechanism influencing macrophage activity [61]. Collectively, these findings underscore the crucial role of circadian regulation in shaping macrophage metabolism and function, offering promising therapeutic avenues for metabolic and inflammatory diseases.

### DCs: modulation of antigen presentation

Upon activation via pattern recognition receptors (PRRs), DCs undergo a rapid metabolic reprogramming characterized by a shift to aerobic glycolysis. This shift fuels the biosynthetic and energetic demands of DC maturation, including membrane expansion, cytokine production, and effective T cell priming. iNOS-mediated nitric oxide production suppresses oxidative phosphorylation (OXPHOS), reinforcing glycolysis as a primary metabolic pathway [62, 63]. In parallel, glycolysis supplies substrates for fatty acid synthesis, while lipid metabolites, such as oxysterols, modulate DC function in a context-dependent manner. During cross-presentation, DCs further integrate glycolysis, OXPHOS, and lipid metabolism to preserve antigens, support peptide loading, and promote efficient MHCⅠ trafficking—processes critically dependent on NADPH oxidase 2 (NOX2)-mediated phagosomal alkalinization, lipid body dynamics, and endoplasmic reticulum (ER) homeostasis [62, 63]. Beyond these classical mechanisms, emerging evidence reveals that circadian rhythms orchestrate DC metabolism and function. Specifically, deletion of the core clock gene *Bmal1* in DCs abolishes time-of-day differences in parasite expulsion and alters the type 1 helper T cell/type 2 helper T cell (Th1/Th2) balance. Circadian-synchronized DCs demonstrate BMAL1-dependent IL-12 responses, linking the clock to metabolic pathways such as amino acid metabolism and the tricarboxylic acid (TCA) cycle [64].

Mitochondria undergo circadian-regulated cycles of fusion and fission, shifting between tubular and fragmented states to modulate ATP production and calcium handling [63]. These morphological transitions are not merely structural—they influence key functional outputs essential for DC activity, such as energy availability and intracellular calcium signaling [42, 63]. Emerging evidence links the molecular clock gene *Bmal1* to circadian rhythms in mitochondrial morphology, calcium oscillations, and antigen processing. Deletion of *Bmal1* disrupts rhythmic antigen presentation and weakens T cell priming. Notably, pharmacological manipulation of mitochondrial dynamics with Mdivi-1, a dynamin 1 like (DNM1L, also named as DRP1) inhibitor promoting mitochondrial fusion, can rescue circadian deficits in antigen processing. These findings highlight a crucial role for circadian-regulated mitochondrial dynamics in optimizing DC function and shaping time-of-day-dependent immune responses [42].

It is worth noting that the fusion/fission cycles of mitochondria exhibit circadian oscillations, as demonstrated in DCs [42], and are not only crucial for energy production but also for immune function, particularly in neutrophils. Mitochondrial fusion, driven by key proteins like mitofusin-2 (MFN2) [65], enhances neutrophil function by promoting ATP production, which in turn supports the formation of NETs [66]. Additionally, mitochondrial integrity is essential for neutrophil motility and tissue extravasation. In both zebrafish and murine models, loss of *Mfn2* results in impaired neutrophil migration, causing these cells to remain trapped in the vasculature [65]. These observations suggest a possible circadian regulation of mitochondrial dynamics in neutrophils, which may influence their trafficking and effector functions. Understanding how mitochondrial architecture and metabolism are linked to circadian rhythms in neutrophils could uncover new insights into the temporal regulation of immune responses and inflammation, offering potential therapeutic avenues for controlling neutrophil-mediated inflammatory diseases.

## Conclusion and future directions

Despite significant progress in understanding the role of circadian rhythms in myeloid cell biology, many questions remain unanswered (Table 1). Future studies could focus on elucidating the molecular mechanisms by which circadian components regulate immune cell functions in various physiological and pathological contexts. Specifically, the interplay between systemic cues (such as neuroendocrine signals and metabolic fluctuations) and intrinsic cellular clocks in myeloid cells warrants further investigation.

**Table 1.** Future research directions: circadian rhythms and myeloid cell function.

Research area	Open questions/future focus
Metabolic regulation	How does the circadian clock regulate glycolysis, oxidative phosphorylation, and lipid metabolism in myeloid cells?
Intrinsic versus extrinsic control	How do cell-autonomous circadian clocks interact with systemic signals (e.g. hormones or feeding) to regulate immune cell function?
Myeloid cells other than monocytes	What are the circadian roles and regulatory mechanisms in eosinophils, basophils, and mast cells?
Circadian disruption effects	How does circadian misalignment (e.g. due to shift work or jet lag) impair myeloid immune functions and contribute to chronic disease?
Chronotherapy	Can we enhance treatment efficacy (e.g. cancer immunotherapy or vaccination) by timing interventions?
Aging and immune rhythmicity	How does aging alter circadian control in myeloid cells? Can restoring clock function rejuvenate immune responses in the elderly?

One promising area of research is the application of chronobiology in metabolic-immunotherapy. Given the diurnal variations of metabolic pathways during antigen presentation, cytokine production, and immune cell trafficking, optimizing treatment schedules for infections, vaccinations, and cancer immunotherapies based on circadian rhythms could enhance therapeutic efficacy. Moreover, understanding how circadian disruption, such as that caused by disturbed eating behaviors, shift work, jet lag, or chronic inflammation, impacts myeloid cell function may reveal novel strategies for mitigating immune-related disorders.

Lastly, while much research has focused on macrophages, neutrophils, and DCs, less is known about the circadian regulation of other myeloid populations, such as eosinophils, basophils, and mast cells. Exploring their rhythmic behaviors and functional consequences could provide valuable insights into allergic diseases and other immune-related conditions.
